# Potential and challenges of specifically isolating extracellular vesicles from heterogeneous populations

**DOI:** 10.1038/s41598-021-91129-y

**Published:** 2021-06-02

**Authors:** Susann Allelein, Paula Medina-Perez, Ana Leonor Heitor Lopes, Sabrina Rau, Gerd Hause, Andreas Kölsch, Dirk Kuhlmeier

**Affiliations:** 1 Fraunhofer Institute for Cell Therapy and Immunology IZI, Leipzig, Germany; 2 Martin Luther University Halle-Wittenberg, Biocenter, Halle (Saale), Germany

**Keywords:** Biological techniques, Cancer, Cell biology, Cancer, Biomarkers, Diagnostic markers

## Abstract

Extracellular vesicles (EVs) have attracted interest due to their ability to provide diagnostic information from liquid biopsies. Cells constantly release vesicles divers in size, content and features depending on the biogenesis, origin and function. This heterogeneity adds a layer of complexity when attempting to isolate and characterize EVs resulting in various protocols. Their high abundance in all bodily fluids and their stable source of origin dependent biomarkers make EVs a powerful tool in biomarker discovery and diagnostics. However, applications are limited by the quality of samples definition. Here, we compared frequently used isolation techniques: ultracentrifugation, density gradient centrifugation, ultrafiltration and size exclusion chromatography. Then, we aimed for a tissue-specific isolation of prostate-derived EVs from cell culture supernatants with immunomagnetic beads. Quality and quantity of EVs were confirmed by nanoparticle tracking analysis, western blot and electron microscopy. Additionally, a spotted antibody microarray was developed to characterize EV sub-populations. Current analysis of 16 samples on one microarray for 6 different EV surface markers *in triplicate* could be easily extended allowing a faster and more economical method to characterize samples.

## Introduction

During the last decade, the interest of the scientific community in extracellular vesicles (EVs) has exponentially grown due to their close relation with their cellular origin^1,2^. Being an important mediator in cell-to-cell communication, EVs deliver cell-specific cargo and surface biomarkers in an organ-specific manner^3^. For this purpose, EVs are continuously released from all cells into all type of bodily fluids^4,5^. The molecular content of EVs, that includes proteins, nucleic acids and lipids, is surrounded by a lipid bilayer. Consequently, the cargo is stabilized and protected from the extracellular milieu conditions making them extremely attractive for the diagnostic purposes using ‘liquid biopsy’^6,7^. This strategy uses circulating biological material derived from an organ of interest in a minimally invasive manner for timely relevant clinical information^8^. Thus, conventional invasive tissue biopsies could be avoided, minimizing the risk of infection and allowing to track information from inaccessible tissues^9,10^.

A variety of EV subpopulations with different nomenclature based on their cellular origin, biogenesis, size, function, cargo or membrane markers are found in the literature^11^. Due to its heterogeneity nature, the International Society for Extracellular Vesicles (ISEV) recommends the use of the general term “Extracellular Vesicles”^12^. EVs are divided into two groups based on their biogenesis: exosomes and microvesicles. Exosomes are 30 to 120 nm in size and are released from multivesicular endosomes (MVE) during fusion with the plasma membrane^13–15^. Microvesicles, on the contrary, are highly heterogeneous in function and size that ranges from 50 nm up to 10 µm. Oncosomes, microvesicles from oncogenic origin, and apoptotic bodies belong to this cluster^16^. Microvesicles are directly shed into the extracellular milieu by budding of the cell membrane^17^. Furthermore, EVs have been found of different densities, from low to high density^5,12,18^ and diverse morphology^19^ contributing to the heterogeneity. Recently, a novel subgroup of vesicles of approximately 35 nm that lack an external membrane was discovered, the so called exomeres^15^. Consequently, EV populations seem to bo more complex than what we know today and subgroups with different characteristics need to be investigated in detail.

There are a variety of enrichment techniques available to study EVs. Some methods rely on size (differential ultracentrifugation (UC), size exclusion chromatography (SEC), ultrafiltration (UF), sequential filtration), density (density gradient (DG) centrifugation), expression of surface proteins (immunoaffinity), solubility change (polymer-based precipitation) or a combination^20^. As studies of different EV techniques might illustrate different outcomes^21,22^, a thoroughfully characterization of the isolated EV fraction is of utmost importance, as long as there are no standardized and reproducible protocols. Therefore, it is recommended that an EV characterization includes: quantification and particle size distribution (nanoparticle tracking analysis, tunable restive pulse sensing), protein analysis (western blot, ELISA, flow cytometry, ExoView) and visualization by electron microscopy^12,23^. However, the intrinsic heterogeneity of EVs and the current limits of analytical equipment, usually intended for cellular research, as well as the low amount of analyte make EV research challenging^24^. In consequence, there have been efforts to promote transparency and to build up reproducibility by the online platform EV-TRACK^25^ and the extensive document “Minimal Information for Studies of Extracellular Vesicles guidelines (MISEV)”^12^.

**Figure 1:**
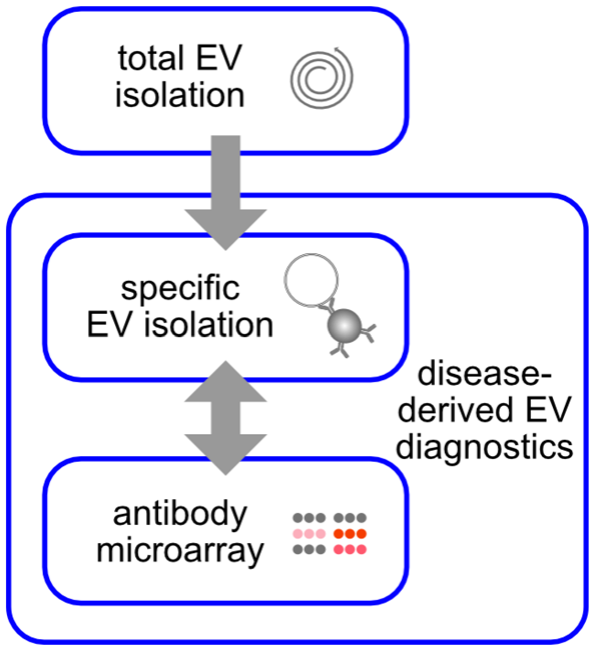
Workflow of the study. Comparing different total EV isolation methods for protein characterization to proceed with surface protein-specific EV capture from cell culture supernatant. The antibody microarray analysis of surface EV marker can serve as a tool to rapidly investigate targets for specific isolation. Selecting tissue- or disease-derived EVs can be applied in EV diagnostics.

The comparison of different EV isolation methods to analyze cell culture supernatant in regard to CD63 expression in relation with purity, yield and size distribution of EVs has revealed the highest EV purity by DG, followed by UC. The least was found for precipitation kits. Additionally, mRNA profiling was equal independent of the method^26^. Similar outcomes concerning yield and purity of the EV fraction were found by Lobb et al. and Tauro et al., showing that a combination of UF and SEC is comparable to DG in terms of purity^21,27^. Furthermore, they found that EpCAM-based immunoaffinity was superior to UC and DG defined by the presence of the EV marker CD9, CD81, TSG101 and ALIX^21^. However, these investigations focused primarily on EV marker but not on cell type-specific proteins which are relevant in tissue-specific EV isolation from liquid biopsy. To date, it is well known, that the cell type and its composition, next to physiological and patho-physiological stimuli, influences the biogenesis and cargo of EVs^4,5^. Therefore, we intended to compare common EV enrichment strategies to find a method that is suitable for an initial comprehensive characterization of EV proteins of benign and malignant prostate-derived EVs from cell culture supernatants. Cell culture supernatants derived from prostate cancer cells were used as a proof-of-concept model. The current prostate cancer diagnosis based on serum PSA levels results in high numbers of false-positive as well as false-negative results and necessitates for novel diagnostic approaches^28–30^. Under healthy basal conditions the prostate specific membrane antigen (PSMA or FOLH1) is found in the cytosol of the cells, while during tumorigenesis PSMA is over-expressed and relocated to the membrane exhibiting an extracellular domain^31,32^. This makes PSMA accessible and a promising candidate for liquid biopsy-based diagnostics. Besides, the ubiquitous EV marker CD9 seems to be overexpressed in prostate cancer^33,34^. Hence, we further investigated if PSMA- as well as CD9-targeted specific isolation of prostate cancer-derived EVs is feasible using immunomagnetic beads. The analysis of EV surface markers as potential targets for specific isolation was performed by an in-house spotted antibody microarray approach to increase sample throughput in a more efficient and cost effective manner (Fig. 1).

## Results

### Comparison of EV enrichment methods

A variety of challenges delays the use of EVs as a diagnostic tool. One of them is the EV release into media containing foreign proteins. To avoid this cross-contamination, serum-free incubation for EV harvest after 24, 30, 48 and 72 h was performed with cellular viability measurement. Overall, the cell viability decreased constantly in all cells deprived of serum. After 30 h incubation time, PC3 and LNCaP cells, showed a viability similar to cells cultured in serum-containing medium, whereas PNT1A and 22Rv1 cells, reached comparable results already after 24 h (Fig. S1).

**Figure 2:**
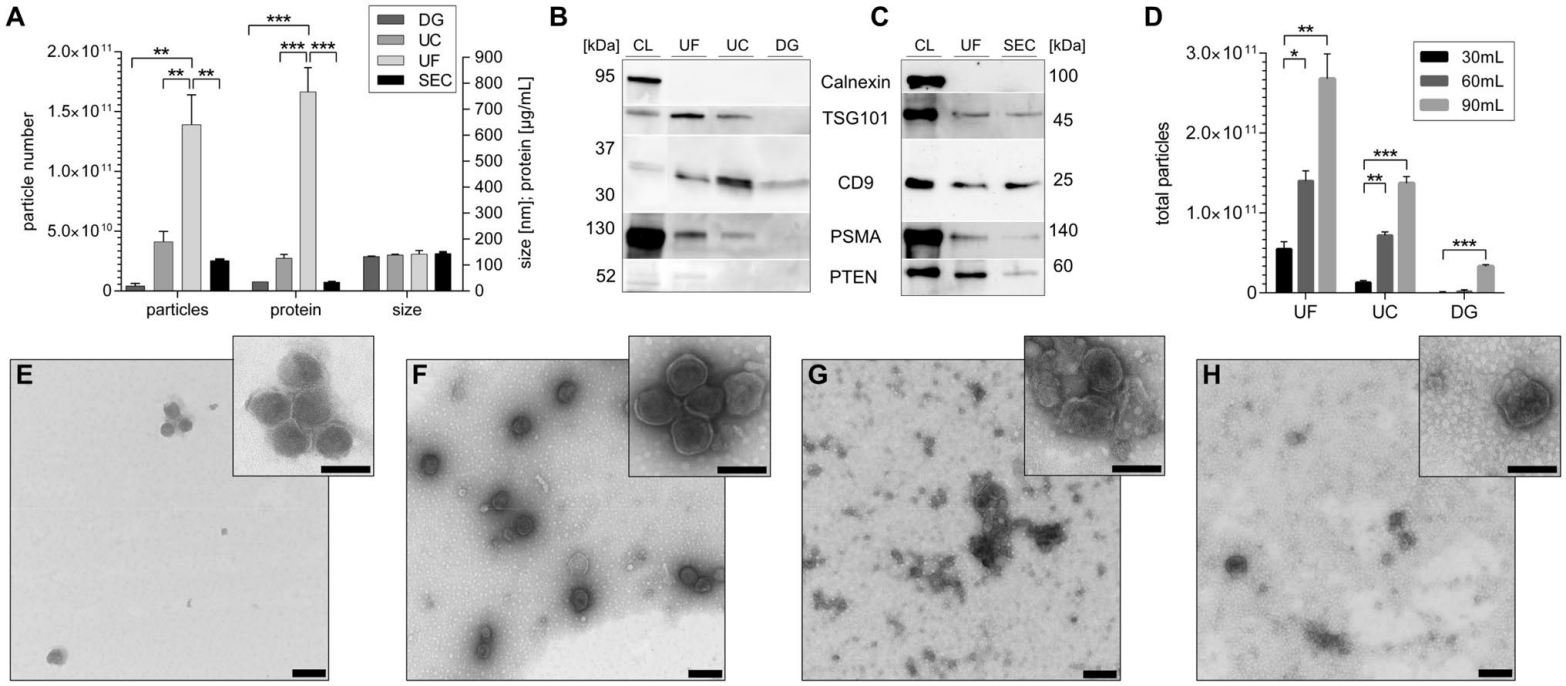
Comparison of EV isolation methods from 22Rv1 cell culture supernatant after 24 h of serum-free incubation. Density gradient centrifugation (DG), differential ultracentrifugation (UC), ultrafiltration (UF) using a MWCO of 100 kDa and its combination with size exclusion chromatography (SEC) analyzed based on NTA data and protein concentration (**A**), western blot of 1E + 9 particles (**B,C**), total particle number per starting volume of supernatant (**D**) and transmission electron microscopy of DG (**E**), UC (**F**), UF (**G**) and SEC (**H**) samples of 16 k fold magnification with scale bars of 200 nm in the big square and 40 k fold magnification with scale bars of 100 nm in the small square (n = 2 ± SD). Statistical differences were analyzed using one-way ANOVA with a P value equal ≤ 0.005 *, 0.01 ** and 0.001 ***.

Due to the absence of standardized methods, we aimed at comparing most common EV preparations to find a suitable method for characterizing prostate-derived EVs. Therefore, density gradient centrifugation (DG), differential ultracentrifugation (UC), ultrafiltration (UF) and a combination of ultrafiltration and size exclusion chromatography (SEC) were performed (Fig. S2). Particle concentration and size distribution determined by NTA (Fig. S3) detected a major peak for the particle hydrodynamic diameter at ~ 110 nm and a mean of ~ 140 nm independently of the isolation method. In regard to the total particle recovery, starting with the same initial supernatant volume and preparation, the lowest particle number was observed after DG of 4.1E + 9, while SEC yielded 2.5E + 10, UC 4.1E + 10 and UF 1.4E + 11 particles. This correlated with the protein concentration showing the least for SEC (33.8 µg/mL) and DG (35.5 µg/mL) and increasing concentration for UC (127 µg/mL) and UF (767 µg/mL) (Fig. 2A). Cellular protein contamination and EV presence was evaluated by western blot of 1E + 9 particles. Calnexin, a non-vesicular protein, was absent, while the EV marker CD9 was detected across all preparations relatively equal, with only slight reduction in UF and DG. TSG101 was found in all preparations, except in DG. PSMA, a protein mainly expressed in prostate tissue, was detected in each preparation with the strongest signal in UF and the faintest signal in DG. On the contrary PTEN, a protein affected in prostate cancer that supports apoptosis and cell cycle arrest via inhibition of the PI3K-AKT-mTOR pathway^35^, was found only in UF and SEC preparations. Relatively similar protein intensities of CD9 and TSG101 were observed for UF and SEC, whereas PSMA and PTEN signals declined in SEC (Fig. 2B,C). As expected, increasing starting volumes resulted in increasing total particle numbers analyzed by NTA, independent of the enrichment method (Fig. 2D).

TEM analysis confirmed the presence of EVs of ~ 100 nm in diameter in all samples. Despite the reduced number, DG showed the highest EV purity in comparison with the other methods (Fig. 2E). UC showed increasing numbers of EVs with lower purity (Fig. 2F). In both preparations EV clusters were found frequently, whereas UF, with the lowest purity, showed clusters of mixed origin, such as EVs entangled in proteins (Fig. 2G). Subsequently, UF samples subjected to SEC reduced the protein background and small EVs (< 100 nm) were visible (Fig. 2H).

### EV protein characterization

**Figure 3:**
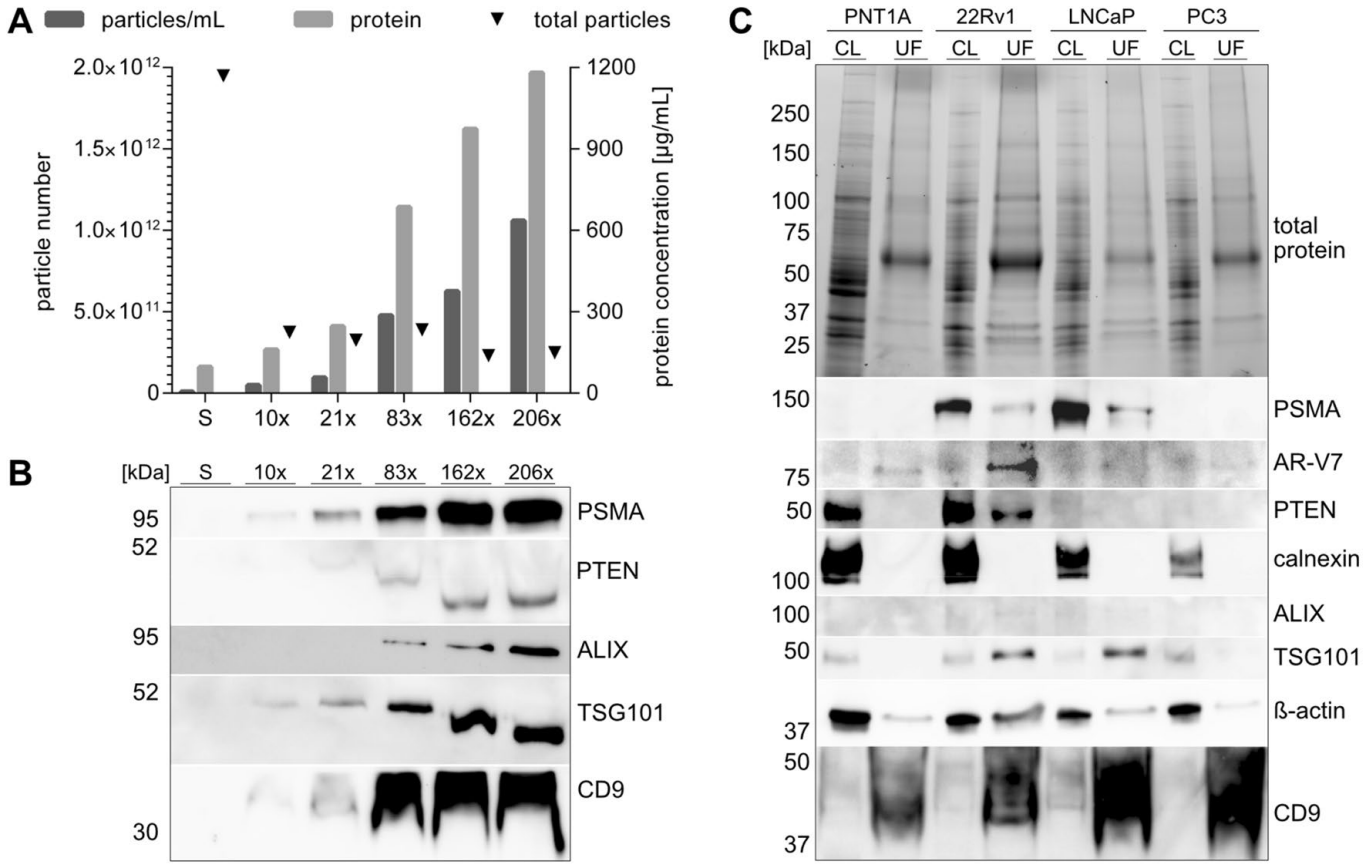
Analysis of increasing concentration of ultrafiltrated 22Rv1 supernatant (S) by NTA, protein concentration (**A**) and western blot (**B**). Characterization of total protein in SDS-PAGE and prostate-related and EV markers in western blot of ultrafiltrated supernatants of 5E+9 particles (UF) and 10 µg protein of cell lysates (CL) from PNT1A, 22Rv1, LNCaP and PC3 cells after serum-free incubation after 24 and 30 h, respectively (**C**).

Comparing the different enrichment methods, the absence of cellular contamination and the most intense signals for the investigated proteins with special focus on PSMA and PTEN lead to the decision on using UF in subsequent experiments to avoid the potential loss of EV subpopulations for characterization. First, the concentration factor by UF with respect to protein concentration and EV marker was evaluated using 22Rv1 supernatant. A proportional increase in particle and protein concentration with rising concentration was observed. Calculation of the total particle revealed a drop of the total particle number after the tenfold sample, while higher concentration factor remained relatively constant (Fig. 3A). The protein expression of prostate- and EV-associated markers showed similar results with a proportional behavior of concentration factor to signal intensity of each protein. The lowest detected PSMA expression corresponded to the lowest concentration factor similar to the classical EV markers TSG101 and CD9. PTEN signals were less intense and started to appear from 21-fold concentration onwards, while ALIX was only visible in the 83-fold concentrated sample (Fig. 3B).

As EVs heterogeneity in size and cargo requires an initial protein characterization, four different prostate cell lines were compared with their respective EV fraction. In general, PNT1A, a non-tumorigenic prostatic cell line, showed less particles in UF from the same starting volume as compared to the evaluated prostate cancer cell lines. This correlated with previous reports that found higher EV numbers from cancer than healthy cells^36^. A similar total protein amount within the cell lysates and EV samples was revealed on the stain-free gel. However, a dissimilar pattern between cell lysates and EV samples was observed with large bands between 50 and 75 kDa in the EV fractions (Fig. 3C).

Moreover, PSMA was absent in PNT1A and PC3, but highly expressed in 22Rv1 and LNCaP cell lysates (CL) and detected in their derived UF samples. Surprisingly, the opposite was true for AR-V7 being highly enriched in UF than in CL, especially in 22Rv1. Another protein associated with prostate cancer is PTEN. Deletion contributes to proliferation, invasiveness and metastasis^37,38^ and it is described as a biomarker candidate for prostate cancer^39^. PTEN was expressed in PNT1A and 22Rv1 cells, while only 22Rv1 EVs showed a PTEN band.

Calnexin was observed only in CL. The general EV markers ALIX, TSG101, beta-actin and CD9 were found to be similarly expressed within the cells. However, for the applied particle number, ALIX is barely visible in UF samples regardless of the cell line, with only 22Rv1 showing a faint band. TSG101 was more intensely in 22Rv1 and LNCaP UF preparations than in the respective CL. No signal for TSG101 was observed for PNT1A and PC3. Beta-actin was detected across all UF samples. Likewise, CD9 was present with the strongest signals within the overall analyzed EV proteins and increasing signals from the healthy control PNT1A to the prostate cancer cell lines 22Rv1, LNCaP to PC3 (Fig. 3C). Most importantly, the data represents the need of EV protein characterization from each cell line, since no conclusion from the CL can be predicted.

### Immunomagnetic isolation

**Figure 4:**
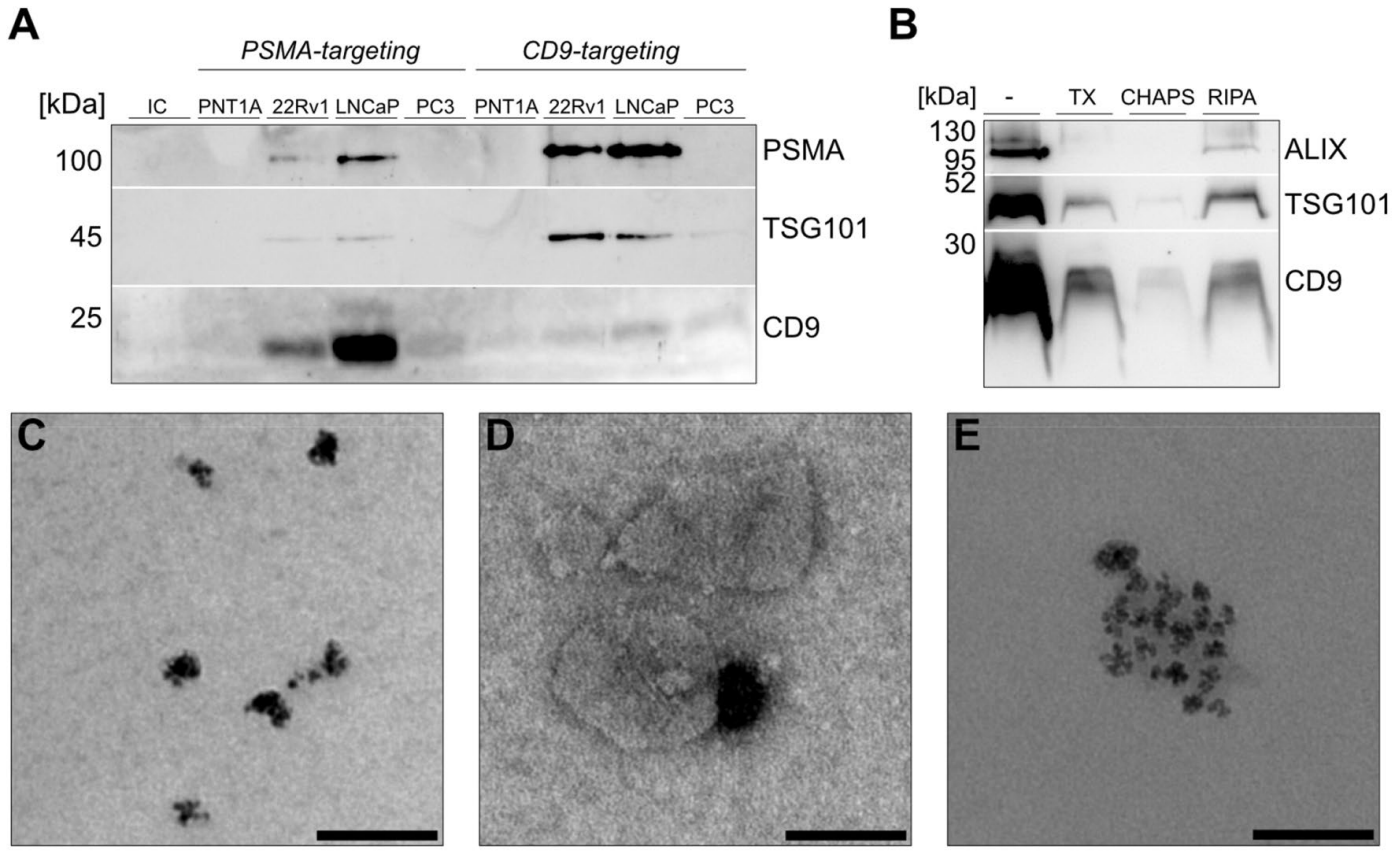
Immunomagnetic bead isolation of EVs targeting PSMA or CD9 from PNT1A, 22Rv1, LNCaP and PC3 ultrafiltrated supernatant after 24 or 30 h serum-free incubation of 2E+10 particles compared to the isotype control (IC) (**A**) and lysis of CD9-targeted EVs from 22Rv1 UF after Triton X-100, CHAPS and RIPA compared to the untreated control (−) by western blot (**B**) TEM of Streptavidin MicroBeads (**C**) and CD9-targeted EVs from 22Rv1 of 50 k fold magnification (**D, E**). Scale bars represent 100 nm (n = 2).

Beside isolating the entire EV population targeting prostate-specific EVs would be inevitable when applying liquid biopsy samples. As a proof-of-concept the surface protein PSMA was investigated. Immunomagnetic pull-down was possible from cell culture supernatant without prior concentration when targeting abundant EV proteins, but as supernatants are quite diluted samples a concentration was beneficial for PSMA-targeting (Fig. S6) as previously advised by other researchers^23^. Therefore, UF as a fast method was applied for pull-down. As expected, PSMA signals displayed in the PSMA-positive cell lines 22Rv1 and LNCaP. The targeted protein should give the most intense signals. However, PSMA appeared to be less intense in samples from PSMA beads. Alike, results for CD9- targeting showed a less prominent CD9 signal than PSMA. To evaluate whether membrane fragments were captured and EV integrity is affected, a luminal EV marker was included in the analysis. TSG101 was detected only in PSMA-targeted EVs from the PSMA-positive cells 22Rv1 and LNCaP and in all cell lines when capturing CD9-positive EVs, except for PNT1A. A markedly stronger expression for TSG101 was found for CD9- compared to PSMA-targeting. Unspecific binding could be excluded by the isotype control incubated with 22Rv1 UF being absent of detectable protein bands (Fig. 4A). TEM confirmed the presence of EVs bound to magnetic beads in the range of ~ 100 nm (Fig. 4C–E). The efficiency of EV lysis on beads was further evaluated using CD9-targeted EVs based on the stronger band intensities in comparison to PSMA pull down. Diminishing signals were found for each lysis method. RIPA buffer-treated samples showed the strongest signals for ALIX, TSG101 and CD9. A reduced detection of ALIX was achieved after Triton X-100 lysis, while CHAPS resulted in the least signal in all tested proteins (Fig. 4B).

### Antibody microarray

**Figure 5:**
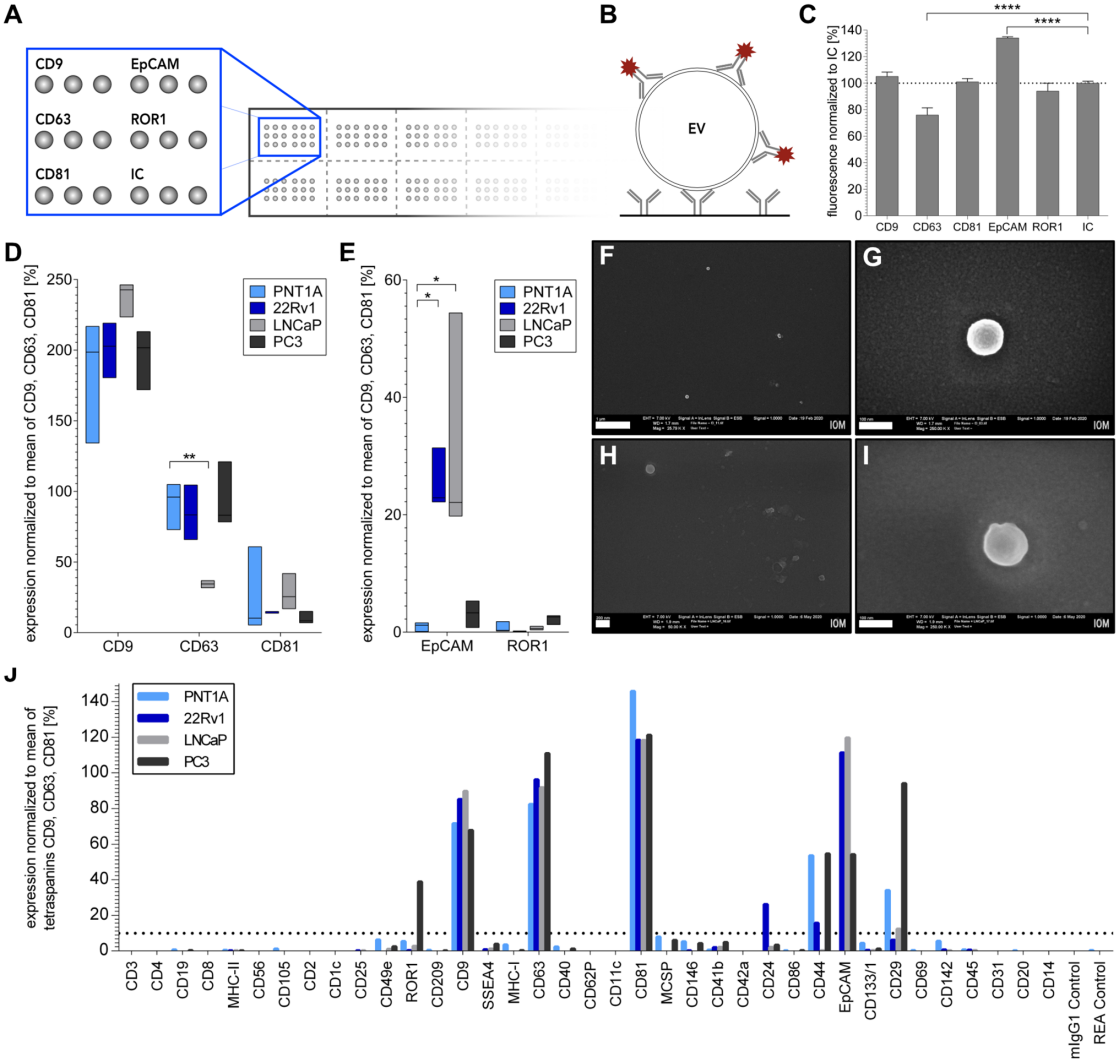
Characterization of EV surface proteins from ultrafiltrated supernatants with an in-house spotted antibody microarray, showing the design of antibodies spots on the functionalized glass slide (**A**) to bind EVs, that will be detected by APC-labeled anti-CD9, -CD63 and -CD81 antibody (**B**). Immobilization of the spotted antibodies observed by Cy3-labeled anti-mouse antibody normalized to the isotype control (IC) (**C**). Results of 1E + 10 particles of UF from PNT1A, 22Rv1, LNCaP and PC3 supernatants after 24 or 30 h serum-free incubation in triplicate of CD9, CD63 and CD81 (**D**) as well as EpCAM and ROR1 captured EVs (**E**) normalized to the mean of all three detection antibodies (n = 3 ± SD). SEM imaging of signal positive spots from 22Rv1 (**F,G**) and LNCaP (**H,I**) of 25 k and 50 k fold magnification with scale bars of 1 µm and 200 nm, respectively (left) and 250 k magnification with scale bars of 100 nm (right). UF from PNT1A, 22Rv1, LNCaP and PC3 after 24 or 30 h serum-free incubation analyzed with the commercially available MACSPlex Exosome Kit (n = 1). Values are normalized to the mean of the detection tetraspanins CD9, CD63 and CD81 (**J**). Statistical analysis was performed using one-way ANOVA with a P value equal ≤ 0.05 *, ≤ 0.01 * and ≤ 0.0001 ****.

According to the MISEV guidelines, a characterization of protein makers needs to be performed to enable the use of the term “EVs”. Thus, in this study, an in-house spotted antibody microarray was developed aiming at detecting EV surface targets that might be helpful to pin point tissue relevant targets for immunomagnetic isolation. The current design comprises antibody spots *in triplicate* for measurements of 6 targets for up to 16 samples on one slide (Fig. 5A). Detection of captured EVs was performed by APC-labeled anti-CD9, -CD63 and -CD81 antibodies (Fig. 5B). The immobilization of the spotted antibodies was observed using a Cy3-labelled secondary antibody against mouse showing comparable immobilization as the isotype control, while only CD63 resulted in reduced levels of 76% and EpCAM in increased values of 134% (Fig. 5C). PSMA could not be detected by the secondary antibody indicating immobilization failure that might possibly occurred due to the different stock buffer containing gelatin. 1E + 10 particles from UF found an increased CD9 expression in all prostate cell-derived EVs (134–246%), with LNCaP giving the highest values. In contrast, CD63 (32–121%) and CD81 (7–61%) were found to be less abundant than CD9 with the lowest expression of CD81 (< 15%) in 22Rv1 and PC3-derived EVs (Fig. 5D). EpCAM, associated with prostate cancer, was upregulated in 22Rv1 (26%) and LNCaP (32%), while PC3 (3%) and PNT1A (1%) showed low levels. ROR1, a tyrosine-protein kinase transmembrane receptor expressed in cancer^40^, was only detectable in PC3-derived EVs (2.2%) (Fig. 5E). In some cases, a high variation within the biological triplicate was observed. However, this observation was not true for all markers of the same cell type: LNCaP showed a high variation for EpCAM while CD9 and CD63 were relatively constant. Afterwards, the microarray was subjected to SEM verifying the presence of EVs as spherical objects of ~ 100 nm (Fig. 5F–I).

Furthermore, to test the detection quality of the developed approach, the commercial MACSPlex Exosome Kit was evaluated in parallel, as normalization is also based on the mean of CD9, CD63 and CD81 values. Similar concentrations for CD9, CD63 and CD81 were observed within all EV samples. CD81 was more present (118–146%) when compared to CD63 and CD9 with signals of 82–111% and 85–90%, respectively. EpCAM (CD326) was prominently found in 22Rv1 (111%) and LNCaP (120%) and to a lesser extent in PC3 (54%), but absent in PNT1A. PC3 (39%) was ROR1 positive (Fig. 5J).

## Discussion

In this study, some of the most common methods for isolation of the overall population of EVs were compared in regard to size distribution, yield, protein concentration, EV associated proteins and with special emphasis on prostate specific proteins. Precipitation-based methods were excluded a priori due to the high protein contamination and uncertainty of EV intactness reported by other researchers^26,27,41^.

All EV preparations showed a similar particle size distribution with a major peak around 110 nm indicating the presence of EVs according to their size. Regarding the total particle amount and protein concentration a positive correlation was found: decreasing yield of particles was associated with decreasing protein concentration. Compared to UF, reduced protein and particle numbers were found for UC, followed by SEC and DG. There are many factors that should be considered. Currently, it is unclear how a high g-force during ultracentrifugation might affect EV stability and function, since EV aggregation after ultracentrifugation has been described^42^. Another aspect is, that our results indicate a higher EV purity after DG followed by SEC or UC compared to UF, as also noticed by TEM. There are still some open questions, for instance how many EVs are lost during the isolation process and which subpopulations are being analyzed. Without a suitable method that quantifies the entire EV population, it is not possible to accurately assess the EV yield after isolation. Despite of this, other groups have proposed the use of fluorescent EV-like particles as spike-in controls to overcome this issue^43–45^. The EV recovery might also be influenced by pellet resuspension after ultracentrifugation, as there is not enough material to obtain a visible pellet. Furthermore, the different biophysical principles isolate subpopulations with different characteristics, affecting protein, but also RNA profiles^46^. This might explain the reduced protein expression of UC and DG preparations for TSG101, PSMA and PTEN, when compared to UF or SEC. Furthermore, the CD9 expression found for each enrichment strategy in a relatively equal manner supports the theory of isolating different subpopulations. PTEN showing decreasing band intensity from UF to SEC have been detected exclusively in the urinary DG EV fraction from prostate cancer patients by LC–MS/MS^47^. The direct release of PTEN has only been shown for a ~ 75 kDa long PTEN-alpha isoform, this is however not the case for the shorter PTEN of ~ 55 kDa^48^, that has been detected in this study indicating its presence in EVs.

Under our lab conditions, ultrafiltration concentrates a volume of 70 mL 100-fold within less than 15 min. SEC needs 15 min for 100 µL of sample, while UC and DG requires 2 h and 22 h, respectively. Another aspect to be aware of is the cost, as an ultracentrifuge exceeds by far the expenses of UF or SEC on medium term prospects. Additionally, staff needs to be trained, due to labor-intensiveness of DG preparation when generating individual gradients by hand making it impractical for diagnostically purpose in the clinics^20^. DG preparation still contains iodixanol, which might interfere with downstream analysis, while the filter material of UF influences the recovery of EVs due to non-specific binding^49,50^. If the main goal is to reach high purity of the EV preparation, DG and SEC are the methods of choice. Therefore, it might be worth considering automated systems. The low loading volumes in both methods can be circumvented by pre-concentration using UF^51^. EVs for diagnostic purposes might reflect, however, the other side of the coin, as high purity seems to be accompanied with the loss of EV subpopulations resulting in a possible loss of crucial information. Additionally, UF is a convenient method with its simple and fast usage together with the possibility to procure a high particle amount in a sample volume that can be adjusted according to the concentration factor being of advantage in particular when using immunomagnetic isolation or the microarray approach.

Immunomagnetic isolation on the contrary aims at targeting a subgroup of the heterogeneous EV population based on one or multiple surface markers. Although a similar TSG101 protein expression was found in UF samples of 5E + 9 particles from 22Rv1 and LNCaP in western blots, TSG101 was moderately expressed in PSMA-targeted EVs compared to CD9-positive EVs. This indicates that 22Rv1 and LNCaP cells release a higher number of CD9-positive than PSMA-positive EVs, consistent with CD9 signals being more intense than PSMA in UF samples in western blot. It remains controversial that CD9 signals were more prominent in PSMA-captured EVs in comparison with CD9-captured EVs. The same behavior was found for CD9 capture, where the PSMA signal was more intense than CD9. One reason might be the presence of luminal PSMA in CD9-positive EVs which could be further investigated using protease-treated versus nontreated EVs. However, similar CD9 signals were detect within the different cell lines implying truncated isoforms due to lysis leading to the missing recognition site for antibody detection. Another explanation might include incomplete RIPA-buffer elution, while EVs were lysed but most of the antibody-targeted surface marker remained on the beads. To prove this hypothesis, different lysis buffers were tested. RIPA and Triton X-100 were the most efficient, but showed less intense signals compared to the untreated control. These preliminary results build a good starting point and give relevant hints for improvement in terms of buffer incubation time, concentration or combination of reagents^52^.

Regarding the characterization after UF from all cell types, no PSMA and low expression of TSG101 in PNT1A and PC3 cell lines were observed, making it difficult to compare those to 22Rv1 or LNCaP cell lines in immunomagnetic isolation. Nevertheless, the use of CD9, with its relative high expression found in all UF samples in western blot, indicate on one hand the presence of EVs in PNT1A and PC3 samples targeting CD9 and on the other hand the specific binding of PSMA-targeting due to missing CD9 signals in PNT1A and PC3 samples.

EV research is a relatively new field with a huge potential despite the already discussed challenges, including the requirement of a profound protein analysis. Although there are common EV markers described in the literature, expression profiles vary within subpopulations depending on the origin and function^18,53^. In this study, none of the UF supernatants showed cellular impurities based on absence of calnexin expression, indicating an adequate viability of the cells under serum starvation conditions. Interestingly, some EV markers such as ALIX were expressed to a lesser extent in prostate cancer-derived cell lines and EVs when compared to TSG101 or CD9. In addition, some cell lines such as PNT1A and PC3 expressed TSG101 and CD9 differentially in EVs as compared to the cell lines 22Rv1 and LNCaP. These results matched previous reports on TSG101 and flotillin expression in PC3 and 22Rv1-derived EVs^53^. It might demonstrate a protein-selective enrichment in EV packaging dependent on the stage of the disease, as EV subpopulation with different cargo and EV proteins specific to prostate cancer has been identified^18,47^. On the other hand, also serum starvation as an external stimuli can change the protein composition of released EVs^54^. The serum-free incubation was, however, applied to all used cell lines. Occasionally a different CD9 size was detected in western blot that might have been the result of glycosylation but also incomplete reduction of disulphide bonds retaining a partially folded structure might be possible.

Consequently, it supports the imperative for an adequate characterization of EVs. The same behavior was observed with TSG101, where a similar expression was detected across all cells lines, but differed in UF samples from the prostate cancer cell lines 22Rv1 and LNCaP. One study examining EV proteins from 60 different cell lines with LC–MS/MS detected ALIX across all samples, while TSG101 and CD9 were present only in two-thirds of the samples^55^. These observations reinforce the importance of evaluating more than one luminal and surface EV marker, since protein amounts below the detection limit of the used method might be misinterpreted as absence or even low EV quality.

Regarding prostate specific proteins, PSMA could be successfully tracked in EV fractions from different cell lines. PSMA was present in a moderate to low manner in EV samples compared to their parental cell lines, confirming the hypothesis as a potential target for liquid biopsy in accordance with the specific capture using immunomagnetic beads. PTEN, however, was found only in the 22Rv1 but not in PNT1A EVs supporting its potential use as biomarker for prostate cancer as described by Gabriel et al.^39^. Moreover, AR-V7 was found more prominently in EV samples than cell lysates. This was especially the case in 22Rv1 UF supernatant, which had the most intense AR-V7 signal. This observation reinforce the potential application of EVs in liquid biopsy, providing information for the diagnostics in prostate cancer. AR-V7 for instance was found to be correlated in castration-dependent prostate cancer with resistance to androgen receptor targeting therapy^56^ providing a possible decision making tool for therapeutics.

Regarding the multiplex approach, both the commercial MACSPlex Exosome Kit and the glass slide microarray showed similar results based on the tetraspanins CD9, CD63 and CD81. The same detection approach was used in another microarray approach for the characterization of EV surface markers by Jørgensen et al.^57^. One decisive factor that should be taken into account is that EV heterogeneity might lead to difficulties in the detection, since this detection approach requires the intact presence of the surface marker of interest and at least one of the tetraspanins. EVs lacking these tetraspanins cannot be detected. Currently, it is unclear how many EVs are actually positive for each tetraspanin and if there is a different expression profile depending on the cell type and stage of disease^36^. An advantage of this labeling strategy is, however, the reduced risk of false positive results when using antibodies instead of a general EV staining dye and higher certainty to detect EVs. Liposomes, for instance, are in the same size range as EVs and lack of the expression of tetraspanins, but can be falsely stained by lipophilic dyes. Until there are no dyes staining exclusively EVs or a protein that is found in all EV subpopulations the detection based on the tetraspanins is preferable.

The expression of the observed tetraspanins in all prostate cell lines was detected on both the glass slide microarray and the bead-based approach of the MACSPlex Exosome Kit. This later method detected tetraspanin levels in the following order from the lowest to the highest expressed marker: CD9 < CD63 < CD81, while the microarray showed a proportional inverse behavior. This might indicate that the used antibodies in the kit and the microarray might target different epitopes or have different affinity with specific binding kinetics towards the target. Therefore, the one-to-one comparison of the results is not appropriate. However, CD9 was found to be the highest in LNCaP-derived EVs and both, EpCAM and ROR1, showed a similar trend in all tested cell lines using both approaches.

The microarray offers a significant advantage due to its overall flexibility to detect EVs from specific tissues, including evaluation of pathological conditions, by targeting surface markers with antibodies or other sorts of affinity molecules related to a certain tissue or disease. A further benefit of the glass slide surface compared to beads is its planar surface which allows to bind EVs directly to the glass surface rather than the irregular structure of the beads in solution targeting EVs from multiple sites. This could lead to a complete saturation of the accessible tetraspanins by the beads and fail or interfere with the detection by the antibodies due to steric hindrance.

## Conclusion

The combination of application and downstream analysis of EVs is an essential factor of guiding the choice of the EV isolation. However, time and workload concerning the amount of processed samples, as well as the available equipment will definitively determine the decision. According to our findings, UF is a convenient method for initial characterization and as a pre-step process in combination with the specific isolation using immunomagnetic beads and to characterize EVs on the antibody microarray. With the later we provide a fast, simple and resource-friendly tool to thoroughly characterize for EV subpopulations with multiplexing up to 33 surface markers *in triplicate* on one sample area and to identify specific surface targets for immunoaffinity isolation. The heterogeneity of EV populations could be investigated with the specific isolation and it might be a favorable technique to obtain disease-specific information from liquid biopsy.

## Materials and methods

### Antibodies

Immunoblotting: PSMA (F-2, 1:1000, Santa Cruz Biotechnologies), PTEN (A2B1, 1:1,000, Santa Cruz Biotechnologies), calnexin (37, 1:1,000), TSG101 (51, 1:1,000, BD Biosciences), beta actin (15G5A11/E2, 1:10,000, ThermoFisher Scientific), CD9 (HI9a, 1:1,000, BioLegend), ALIX (3A9, 1:1,000, Cell Signaling), TSG101 (EPR7130(B), 1:1,000), ARV7 (EPR15656, 1:1,000, Abcam), PSMA (D4S1F, 1:1,000), CD9 (D3H4P, 1:1,000), beta-actin (3E5, 1:1,000, Cell Signaling Technology), HRP-conjugated anti-mouse and anti-rabbit antibody (Dianova, 1:10,000).

Immunomagnetic bead isolation: biotinylated CD9 (HI9a), PSMA (LNI-17) or the isotype control antibody (MOPC-21, BioLegend).

Spotting: CD9 (HI9a), CD63 (H5C6), CD81 (5A6), ROR1 (2A2), the isotype control (MOPC-21, BioLegend), EpCAM (VU-1D9, ThermoFisher Scientific).

Detection on the microarray: APC-labelled CD9 (HI9a), CD63 (H5C6), CD81 (5A6, BioLegend), Cy3-labelled anti-mouse IgG1 antibody (1:5,000, Dianova).

### Cell culture and EV enrichment

PNT1A (Sigma Aldrich), 22Rv1, LNCaP and PC3 (ATCC) prostate cell lines were cultivated in RPMI (Gibco) and Ham´s F-12 (Gibco), respectively, supplemented with 10% fetal calf serum (FCS) and 1% Penicillin/Streptomycin in a humidified atmosphere at 37 °C and 5% CO_2_.

For EV isolation, cells were grown in 175 cm^2^ flasks until ~ 80% confluence, rinsed twice with PBS and grown in 30 mL FCS-free media. After 24 h (22Rv1, PNT1A) or 30 h (LNCaP, PC3) with viabilities > 95%, the supernatants were collected, centrifuged 10 min at 300×*g* and filtered with a 0.22 µm PES bottle top filter (Merck Millipore). For differential ultracentrifugation (dUC), the supernatant was centrifuged 20 min at 2000×g, 4 °C followed by a 2 h centrifugation step at 110,000×*g*, 4 °C in a Surespin 630 rotor in a Sorvall WX ultracentrifuge (ThermoFisher Scientific) to pellet EVs. Iodixanol density gradient centrifugation (DG) supernatants were treated as dUC followed by a top to bottom density gradient approach using OpitPrep (Progen). Four layers of ice cold 4 mL 40%, 4 mL 20%, 4 mL 10% and 3.5 mL 5% iodixanol in DG buffer (0.25 M sucrose, 1 mM EDTA, 10 mM Tris–HCl, pH 7.4) were pipetted into a Senton tube and 0.5 mL sample on top. After 18 h of centrifugation with maximal acceleration and minimal deceleration to avoid gradient disturbance at 100,000×*g*, 4 °C 1 mL fractions were collected. The EV containing fractions 7 to 10 were pooled according to the observed CD9 and TSG101 expression (Fig. S3), filled with 31 mL PBS and centrifuged 3 h at 100,000×*g*, 4 °C. The EV-containing pellets were resuspended in PBS. Ultrafiltration (UF) was performed with Centricon-70 centrifugal filters of 100 kDa MWCO (Merck Millipore) after washing twice with 30 mL PBS with 0.1% Tween 20 (PBST). The filtered supernatant was processed by centrifugation at 1500×*g*, 4 °C with a volume-dependent centrifugation time. The retentate was collected by a 2 min up-side-down spin at 1000×*g*, 4 °C.

Size exclusion chromatography (SEC, IZON, 35 nm) was conducted according to the manufacturer's recommendations. In brief, 0.5 mL of UF samples were applied followed by 0.5 mL PBS steps. Fraction 7 and 8 were pooled for EV collection. All EV samples were aliquoted and stored at − 80 °C.

Immunomagnetic bead isolation of EVs was performed with Streptavidin MicroBeads (Miltenyi Biotec). 2E + 10 particles from UF were incubated with 1.5 µg biotinylated antibodies against CD9, PSMA or the isotype control for 1 h at room temperature (RT) following the addition of 50 µL MicroBeads for 1 h at RT under constant agitation. µColumns (Miltenyi Biotec) were activated by 50 µL of 70% ethanol and equilibrated with 500 µL PBST. Samples were added and washed four times with 100 µL PBST on µColumns placed in a separator to remove unbound material from the beads. Elution was achieved by lysis of EVs by applying 15 µL of 1% Triton X-100, RIPA-buffer (25 mM Tris–HCl, 150 mM NaCl, 1% Triton X-100, 0.5% Sodium Deoxycholate, 0.5% SDS, pH 7.6) or 1% CHAPS for 2 min. The first eluate was discarded, the following three elutions were pooled and analyzed in western blot.

### Protein quantification assay

Cell lysates and EV samples were analyzed for protein content by Pierce BCA Protein-Assay and Qubit Protein Assay Kit (both ThermoFisher Scientific) according to the manual.

### Nanoparticle tracking analysis (NTA)

Samples diluted in PBS were measured thrice for 60 s using the NanoSight LM-10 (Malvern Instruments Ltd.) and the NanoSight NTA 3.0 software on a stable table TS-140 at 25 °C. In the capture mode the camera level was set to 13 and the gain to 3.9, detection threshold was set to 5 for analysis. The particle number served for normalization in subsequent experiments.

### Western blotting

Samples boiled for 10 min at 70 °C in 6 × Laemmli buffer (0.375 M Tris–HCl, 0.6 M DTT, 60% glycerol, 12% SDS, 0.06% Bromphenol blue) were separated in 12% SDS-PAGE or 4–20% stain-free gels (Bio-Rad Laboratories) in Towbin buffer (0.025 M Tris-base, 0.192 M glycine, 0.1% SDS pH 8.6) in a Mini-Protean Tetra Vertical Electrophoresis Cell (Bio-Rad Laboratories). On a Trans-Blot SD semi-dry transfer cell (Bio-Rad Laboratories) a sandwich of extra thick Whatman paper and a nitrocellulose membrane (GE, 0.2 µm) both soaked in anode buffer (48 mM Tris-base, 39 mM glycine, 30% methanol) were placed followed by the gel and another extra thick Whatman paper soaked in cathode buffer (48 mM Tris-base, 39 mM glycine, 20% methanol, 0.05% SDS). Transfer was performed under 25 V for 45 min. The membrane was blocked in 5% BSA in PBST for 1 h at RT. According to the molecular weight the membrane was cut to allow the incubation of more than one primary antibody diluted in blocking buffer over night at 4 °C under constant agitation. After three 5 min washing steps in PBST at RT, the membrane was incubated with the secondary HRP-conjugated antibody for 1 h at RT. Two washing steps for 5 min each with PBST and PBS were performed before the ECL substrate (Bio-Rad Laboratories) was applied and proteins were detected in the ChemiDoc MP device. Raw data in Supplementary Figs. S7–S10.

### Semiquantitative FACS EV analysis (MACSPlex Exosome Kit)

For screening of surface markers the MACSPlex Exosome Kit (Miltenyi Biotec) was used. Single experiments were performed by using 5E + 9 particles in 120µL of UF supernatant in a filter plate according to the manufacturer’s instructions. The FACS Canto II (BD Biosciences) using the FACSDiva 8.0.1 software (BD) was initialized with setup beads prior sample measurement of a minimum of 10,000 events using the blue and red laser. Median fluorescence values were background values subtracted and normalized to the mean of all three tetraspanin medians as described in the manual.

### EV antibody microarray

An in-house antibody microarray was spotted using the sciFLEXARRAYER S3 dispensing system (Scienion) on 3D-Epoxy-Polymer-coated glass slides (PolyAN). Droplet size was set to 2.5 nL with an antibody concentration of 100 µg/mL with the addition of 5% glycerol. The slide was immobilized in the spotting chamber over night and subsequently assembled with a ProPlate Multi-Well Chamber (Grace Bio-Labs) providing 16 samples per slide.

After incubation with blocking solution (PolyAn) for 1 h at RT 1E + 10 particles from UF were added and incubated for 1 h at RT. From this step onwards all incubation and washing steps were performed at RT and under constant agitation. After three washing steps with PBST for 5 min, detection antibody mix of 5 µL each of human anti-CD9/CD63/CD81-APC in total 100 µL PBST was added and incubated for 1 h. Following three wash steps with PBST each 5 min and one washing step with PBS, the slide was dried under nitrogen gas stream. The slide was scanned in the GenePix 4200A microarray reader (Molecular Devices) with a gain set to 400 and a power set to 90%. The generated *.GAL file from spotting was used for analysis in the GenePix Pro software. PBST, ultrafiltrated RPMI and F12-K media served as controls. Successful antibody spotting was observed by applying a Cy3-labelled anti-mouse antibody for 1 h at RT.

### Scanning electron microscopy (SEM)

Microarray slides after EV characterization were fixed using 3.7% glutaraldehyde in PBS for 1 h at RT for SEM. After washing twice with PBS, the sample was dehydrated using sequential steps of 40, 60, 80 and 98% ethanol for 10 min each followed by air drying for 3 h. The slides were sputtered with a 5–15 nm aluminum layer using magnetron sputter deposition in vacuum prior visualization. Images were recorded with acceleration voltages of 7 kV in a SEM Ultra 55 (Carl Zeiss) using the smart SEM software.

### Transmission electron microscopy (TEM)

TEM was performed by EM 900 and a Libra 120 (Zeiss Microscopy GmbH) of 80 and 120 kV, respectively. 3 µL of sample were pipetted on formvar coated electron microscopy cooper grids (200 mesh, Plano GmbH). After three H_2_0 wash steps, 2% uranyl acetate in H_2_0 for negative staining followed, each step for 1 min at RT. Excess liquid was removed and drying was allowed prior to imaging.

### Statistical analysis

Data visualization and analysis was done with GraphPad Prism 9 (GraphPad Software, San Diego). Data were shown as mean ± standard deviation (SD). Statistical significant differences were assessed by one‐way ANOVA with *P* value < 0.05 considered as significant.

### EV‐TRACK

We have submitted all relevant data of our experiments to the EV‐TRACK knowledgebase (EV‐TRACK ID: EV210153)^25^.

## Supplementary Information

Supplementary Information.

## Data Availability

Datasets used and/or analyzed during the current study are available from the corresponding author on reasonable request.
