# Supplementary material for: Potential and challenges of specifically isolating extracellular vesicles from heterogeneous populations
Source: Sci Rep. 2021 Jun 2;11:11585. doi: 10.1038/s41598-021-91129-y (PMC8172572; doi:10.1038/s41598-021-91129-y)
Supplement: Supplementary file 1 — Supplementary Information. [file 41598_2021_91129_MOESM1_ESM.pdf]

Potential and challenges of specifically isolating extracellular vesicles from heterogeneous populations

Susann Allelein*[1], Paula Medina-Perez[1], Ana Leonor Heitor Lopes[1], Sabrina Rau[1], Gerd Hause[2], Andreas Kölsch[1] and Dirk Kuhlmeier[1]

[1]Fraunhofer Institute for Cell Therapy and Immunology IZI, Leipzig, Germany

[2]Martin Luther University Halle-Wittenberg, Biocenter, Halle (Saale), Germany

Corresponding author: susann.allelein@izi.fraunhofer.de

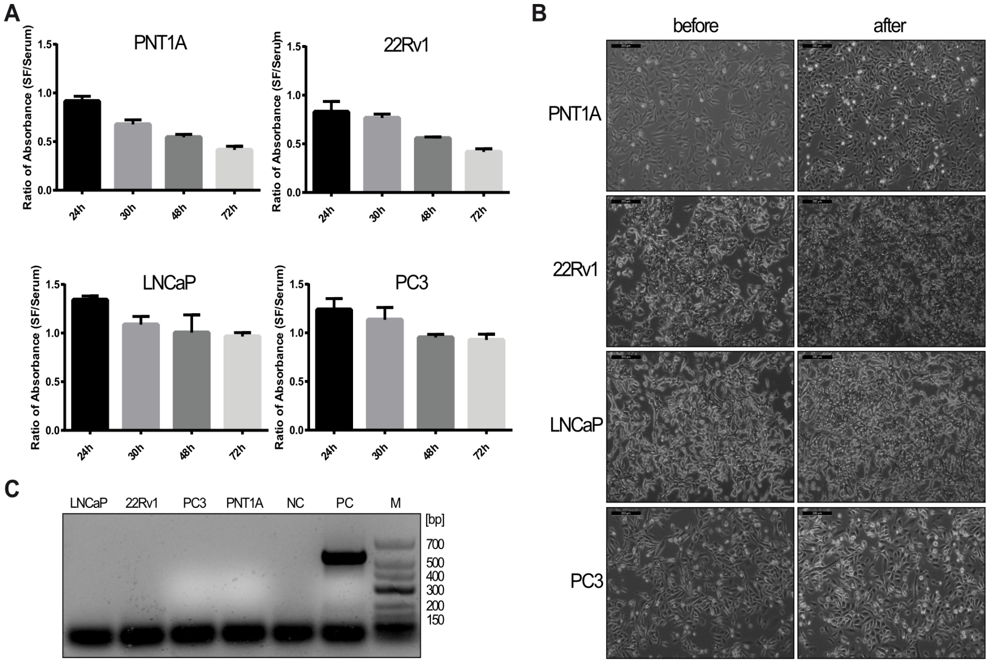

Fig. S1: *Viability analysis after 24, 30, 48 and 72h serum-free incubation (n=3 ± SD) (**A**), microscopic images before and after serum-free incubation (**B**) and PCR analysis for Mycoplasma contamination (**C**) of PNT1A, 22Rv1, LNCaP and PC3 cells compared to a negative (NC) and positive control (PC).*

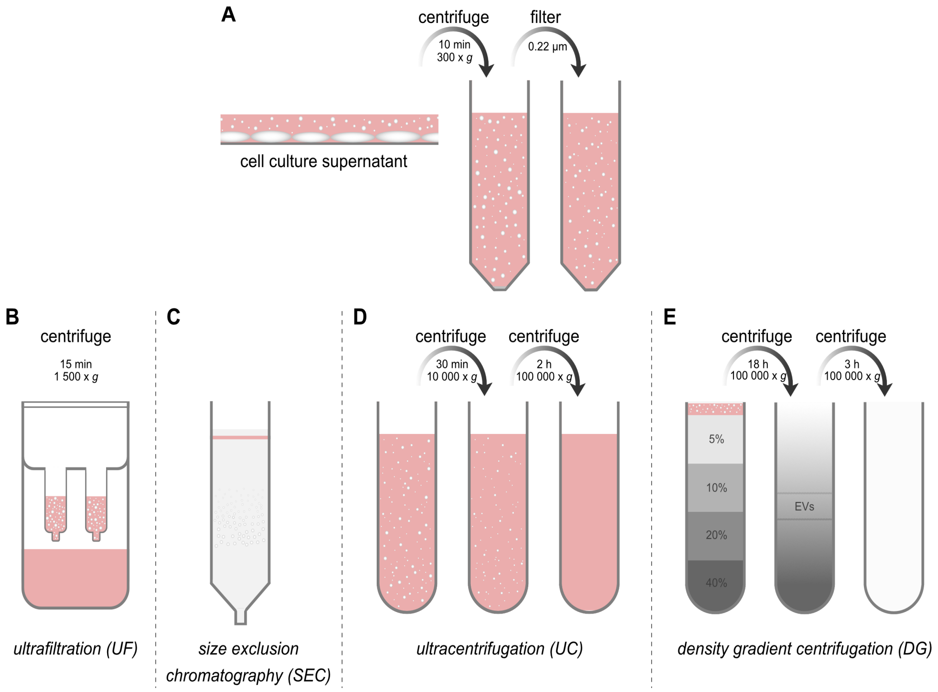

Fig. S2: *Schematic overview of the experimental steps of the most common overall EV isolation methods. Cell culture supernatant is harvested after an appropriate incubation time and processed in order to remove cell and debris before (**A**) ultrafiltration (UF) (**B**), size exclusion chromatography (SEC) (**C**), differential ultracentrifugation (UC) (**D**) or density gradient centrifugation (DG) (**E**).*

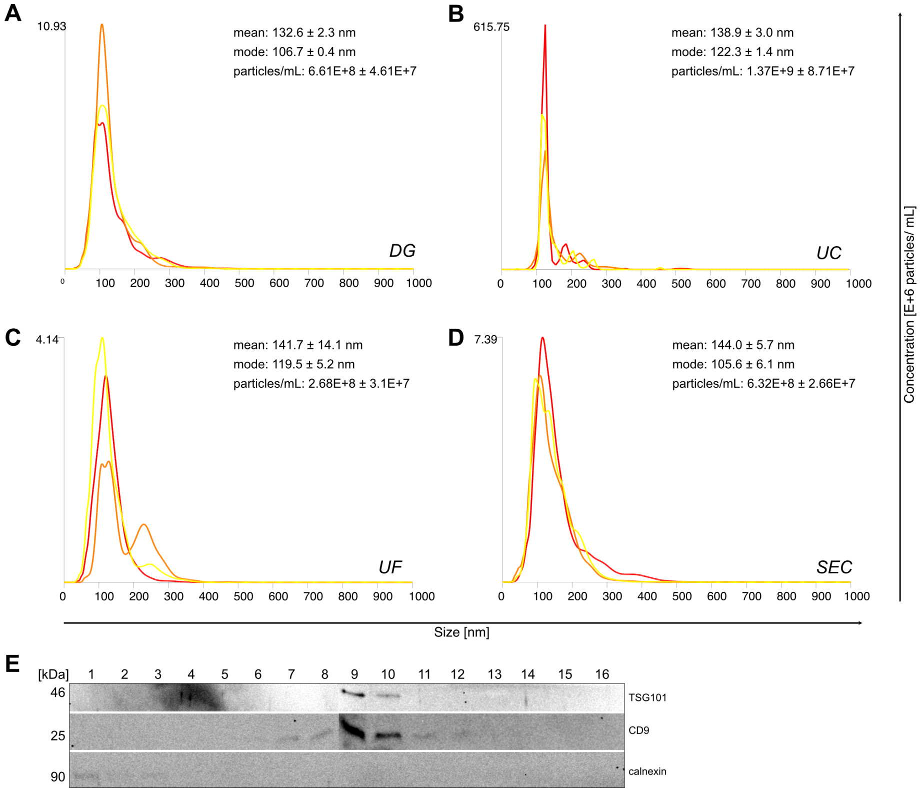

Fig. S3: *Nanoparticle Tracking Analysis of density gradient centrifugation (**A**), ultracentrifugation (**B**), ultrafiltration (**C**) and size exclusion chromatography (**D**) from 22Rv1 supernatant diluted in PBS and western blot analysis of density gradient fractions 1 to 16 for calnexin, TSG101 and CD9 (**E**).*

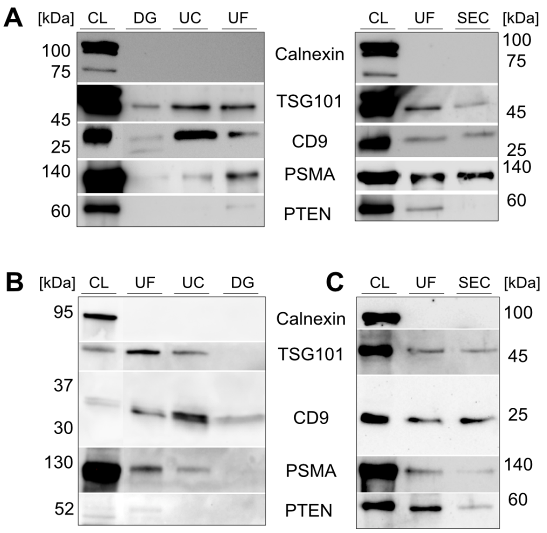

Fig. S4: *Comparison of EV isolation methods from 22Rv1 cell culture supernatant after 24 h of serum-free incubation. Density gradient centrifugation (DG), differential ultracentrifugation (UC), ultrafiltration (UF) using a MWCO of 100 kDa and its combination with size exclusion chromatography (SEC) analyzed Western blot of 1E+9 particles from two different biological replicates. One replicate not show in the main text (**A**) in comparison to the one shown in the main text (**B, C**).*

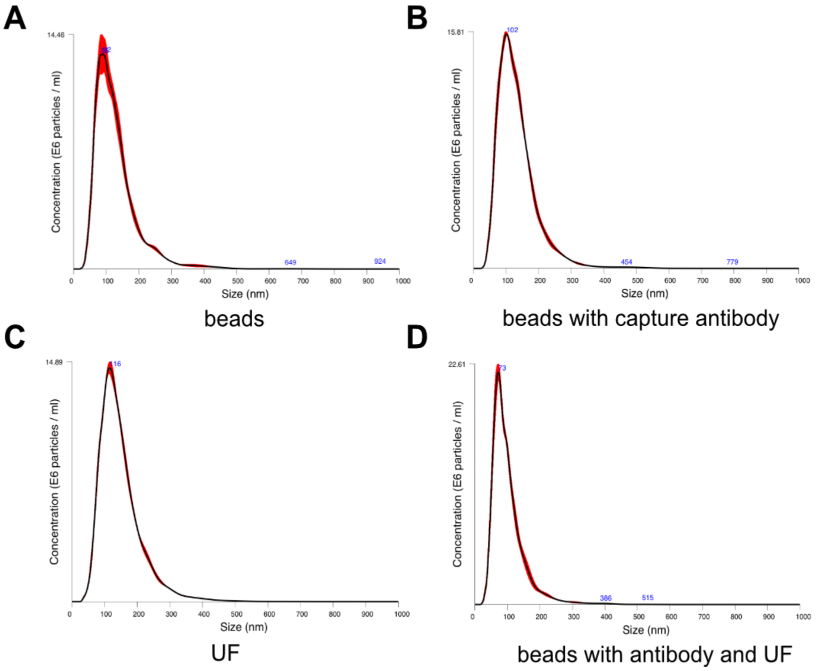

Fig. S5: *Immunomagnetic isolation of 22Rv1-derived EVs. NTA analysis of Streptavidin MicroBeads (**A**), Streptavidin MicroBeads after CD9 antibody incubation (**B**), the sample UF from 22Rv1 cell culture supernatant (**C**) and the bead-antibody-EV-complex after incubation of Streptavidin MicroBeads with the CD9 antibody and the UF sample from 22Rv1 (**D**).*

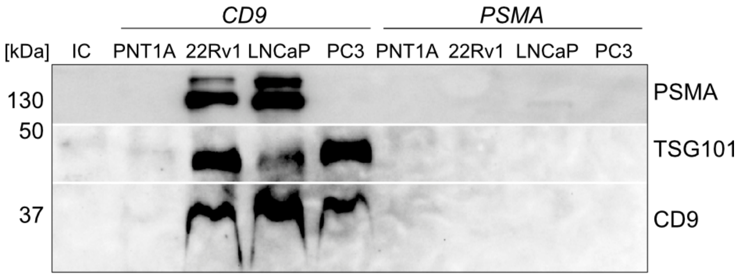

***Fig. S6:** Immunomagnetic isolation of CD9-positive and PSMA-positive EVs from 4mL fresh cell culture supernatant after 24h or 30h serum-free incubation of PNT1A, 22Rv1, LNCaP and PC3 cells after removal of cellular contaminations using 10 min 300 xg and 0.22 µm filtration. Analysis by western blot for PSMA, TSG101 and CD9 in comparison of the isotype control (IC) using 22Rv1 cell culture supernatant.*

Western blot for Fig. 2

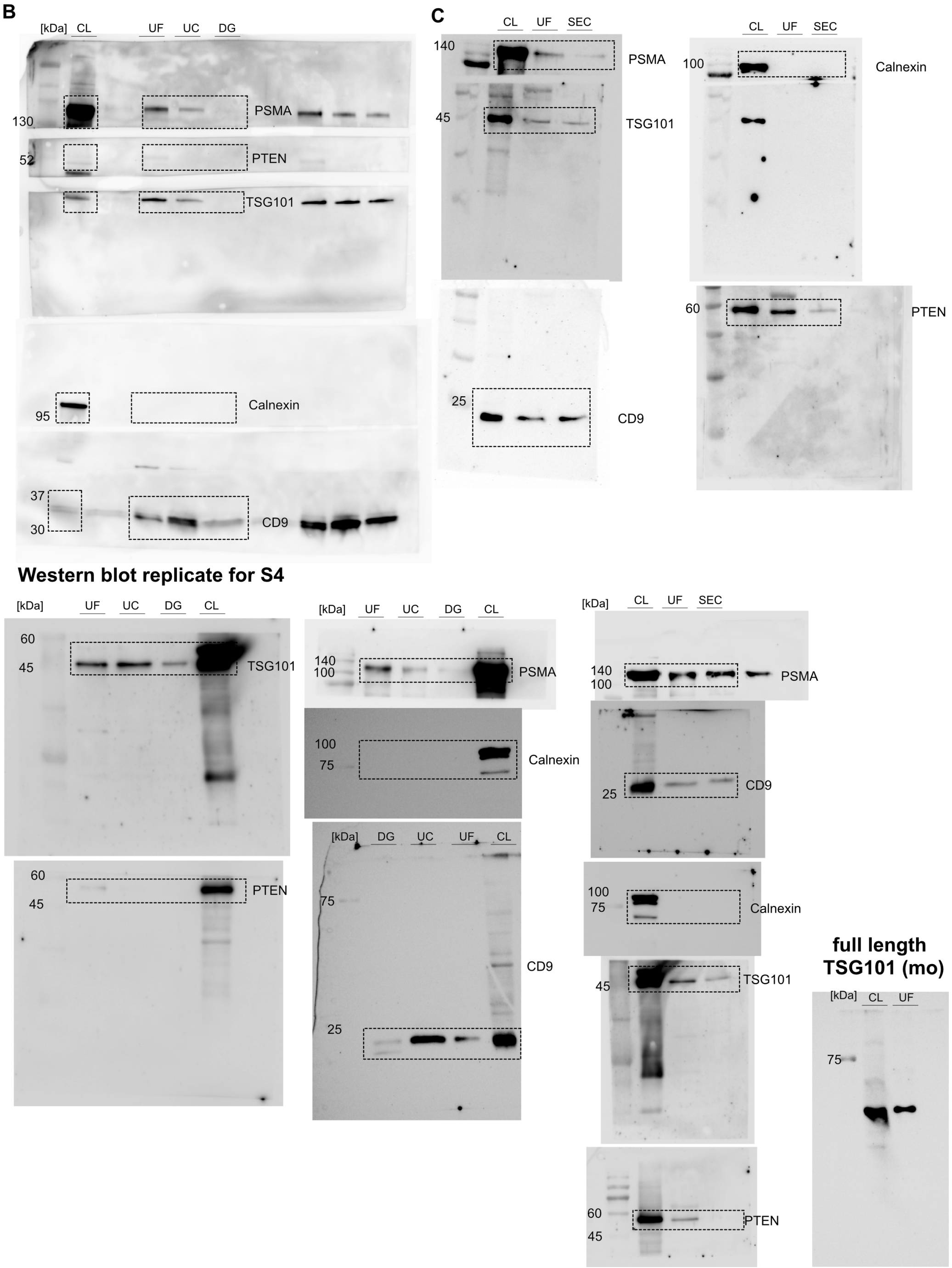

Fig. S7: *Comparison of EV isolation methods. Western blot images with cropped areas indicated by dashed lines. Antibodies were tested previously on full length membranes using UF and CL samples as shown for TSG101 antibody prior to sample testing on membrane cuts according to the molecular weight of the investigated protein. Based on the small amount of material obtained from EV isolation it allowed the investigation of several proteins.*

Western Blot for Fig. 3

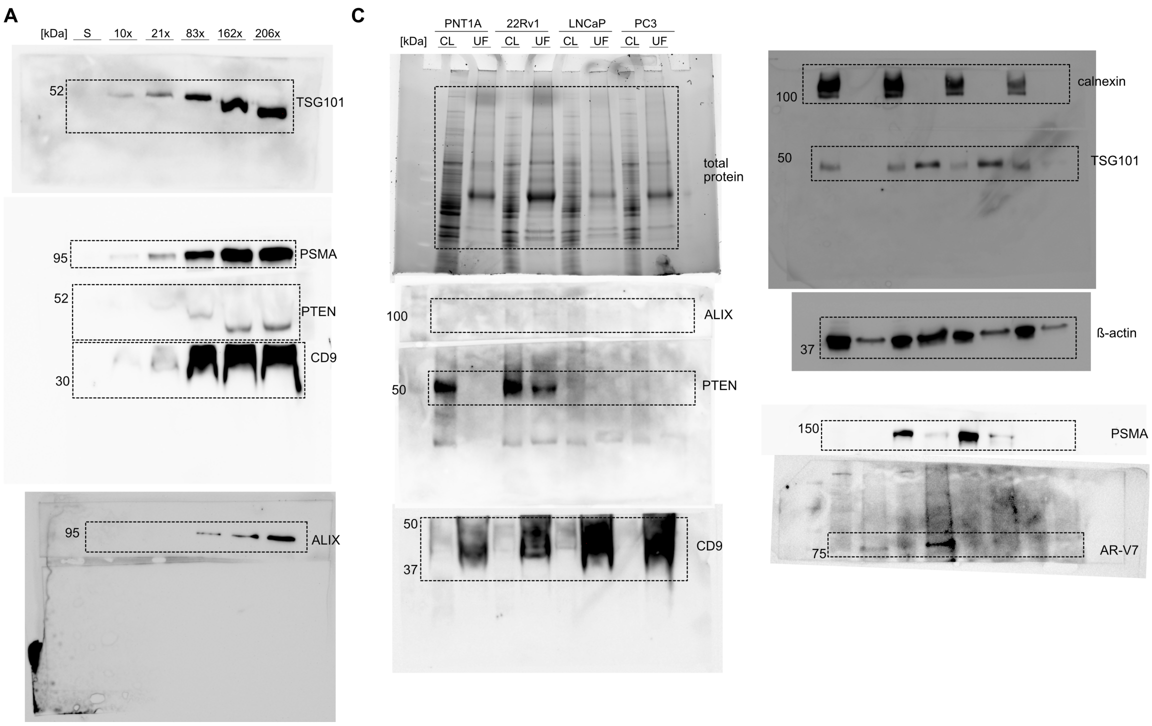

Fig. S8: *Analysis of increasing concentration of ultrafiltrated 22Rv1 supernatant and the characterization of ultrafiltrated supernatants. Western blot images with cropped areas indicated by dashed lines. Antibody incubation on membrane cuts according to the molecular weight of the investigated protein*

Western blot for Fig. 4

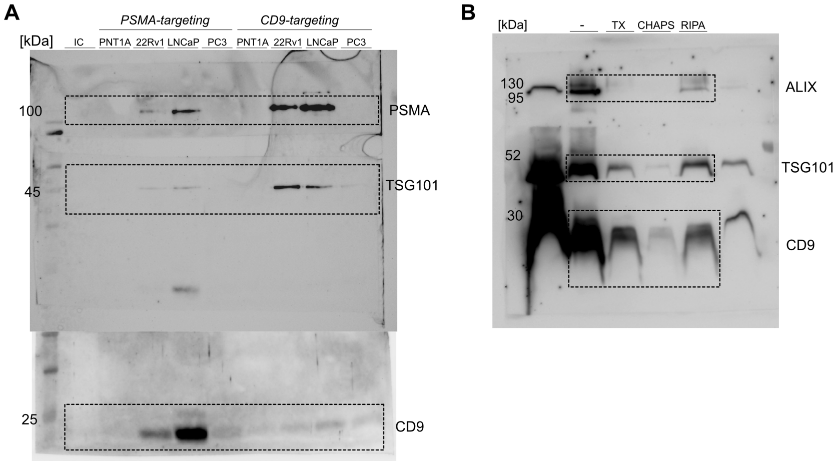

Fig. S9: *Immunomagnetic bead isolation of EVs targeting PSMA or CD9 from PNT1A, 22Rv1, LNCaP and PC3 ultrafiltrated supernatant. Western blot images with cropped areas indicated by dashed lines. Antibody incubation on membrane cuts according to the molecular weight of the investigated protein.*

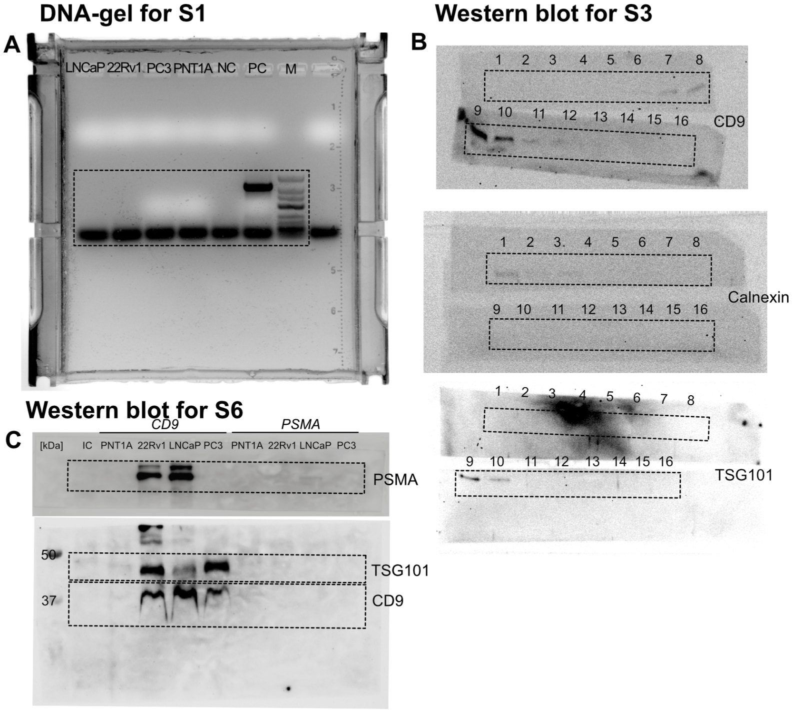

Fig. S10: *Full length DNA gel from S1 (**A**), Western blot images from fraction 1 to 16 after density gradient centrifugation of 22Rv1 supernatant from S3 (**B**). Western blot images from immunomagnetic isolation from cell culture supernatant from S6 (**C**). Antibody incubation on membrane cuts according to the molecular weight of the investigated protein. Cropped areas indicated by dashed lines.*
